# Implementation of the ProMisE classifier and validation of its prognostic impact in Brazilian endometrial carcinomas

**DOI:** 10.3389/fonc.2024.1503901

**Published:** 2024-12-13

**Authors:** Diocésio Alves Pinto Andrade, Murilo Bonatelli, Flávia Escremim de Paula, Gustavo Noriz Berardinelli, Gustavo Ramos Teixeira, Monise Tadin dos Reis, Flávia Fazzio Barbin, Carlos Eduardo Mattos da Cunha Andrade, Vinicius Pereira Aguiar, Alejandro Delfos Hermoza, Welinton Yoshio Hirai, Ronaldo Luís Schmidt, Rui Manuel Reis, Ricardos dos Reis

**Affiliations:** ^1^ Oncoclínicas&CO, Medica Scientia Innovation Research (MEDSIR)/MedSir, Ribeirão Preto, São Paulo, Brazil; ^2^ Molecular Diagnostic Laboratory, Barretos Cancer Hospital, São Paulo, Brazil; ^3^ Pathology Laboratory, Barretos Cancer Hospital, São Paulo, Brazil; ^4^ Barretos School of Health Sciences Dr. Paulo Prata, FACISB, São Paulo, Brazil; ^5^ Gynecologic Oncology Department, Barretos Cancer Hospital, São Paulo, Brazil; ^6^ Gynecologic Oncology Department, Patrocinio Cancer Hospital, Minas Gerais, Brazil; ^7^ Department of Epidemiology and Biostatistics, Barretos Cancer Hospital, São Paulo, Brazil; ^8^ Department of Surgical Oncology, Lagarto Unit, Barretos Cancer Hospital, Sergipe, Brazil; ^9^ Molecular Oncology Research Center, Barretos Cancer Hospital, São Paulo, Brazil; ^10^ Life and Health Sciences Research Institute (ICVS), School of Medicine, University of Minho, Braga, Portugal

**Keywords:** endometrial cancer, molecular classification, ProMisE, biomarkers, Latin-America

## Abstract

**Purpose:**

Molecular classification of endometrial cancer (EC) has emerged as a key approach to individualize therapy and define prognostic outcomes. This study aimed to implement the traditional ProMisE classification in a Brazilian population, compared with a molecular setting of ProMisE biomarkers, and evaluate its impact on patients’ prognosis.

**Patient and methods:**

A prospective cohort of 114 patients with primary EC treated at Barretos Cancer Hospital (BCH) between October 2020 and December 2022 was conducted. Pathology diagnosis, staging, treatment, and follow-up data were collected. The traditional ProMisE methodology was carried out by *POLE* hotspot sequencing and immunohistochemistry (IHC) for p53 and mismatch repair (MMR) proteins. We further evaluate the MMR and *TP53* status by molecular approach, namely microsatellite instability (MSI) by PCR-based and *TP53* mutation analysis by next-generation sequencing (NGS). The results of the 4 molecular groups in both methodologies were compared regarding agreement accuracy and survival outcomes.

**Results:**

Among the 114 cases, the traditional ProMisE groups were: *POLE*mut 15.8%, MMRd 28.1%, p53abn 27.2%, and no specific molecular profile (NSMP) 28.9%. Considering the molecular classification approach, we observed a *POLE*mut group *of 15.8%*, MSI group of 23.7%, *TP53* mutation of 27.2%, and NSMP of 33.3%. The concordance rate of both approaches was 86.8% (99/114 cases) with an overall accuracy of 0.87. Importantly, both traditional and molecular ProMisE approaches were associated with significant distinct overall survival (OS) and progression-free survival (PFS) outcomes, with *POLE*mut patients exhibiting a better prognosis (93.8% OS, at 24 months), whereas the p53abn having a worse survival time (68.9% of OS, at 24 months).

**Conclusion:**

We reported for the first time the Brazilian profile of the ProMisE classification of endometrial cancer and demonstrated the prognostic impact of the traditional and molecular ProMisE classification on patient outcomes.

## 1 Introduction

Endometrial cancer (EC) is the 6^th^ most prevalent neoplasm among women worldwide, representing 4.5% of cancer diagnoses in females. There are an estimated 420,000 new cases per year, and just over 97,000 women will die from this neoplasm in the same period (1). In Brazil, EC is the 7^th^ most prevalent tumor among women, with approximately 8,000 new diagnoses per year (2). Although the absolute numbers are not that impactful, a significant increase in new cases was observed in Brazil, probably related to the rise in obesity rates (3).

For years, the understanding of the pathophysiology of EC was based on the dualistic model proposed in the early 1980s, classifying it into type I (endometrioid) and type II (non-endometrioid) (4). In 2013, with the publication of The Cancer Genome Atlas (TCGA), this neoplasm was molecularly classified into four groups with different prognoses: *POLE* ultramutated (*POLE*), microsatellite instability hypermutated (MSI), copy-number low (endometrioid), and copy-number high (serous-like) (5). Due to a complex multi-omics methodology, it was difficult to reproduce and implement in daily practice.

Subsequently, two independent groups developed more straightforward methods to classify EC molecularly. The Proactive Molecular Risk Classifier for Endometrial Cancer (ProMisE) and Leiden/TransPORTEC classification used immunohistochemistry to detect the presence/absence of mismatch repair (MMR) proteins and to evaluate p53 expression, using, in both methodologies, the molecular analysis of *POLE* hotspot mutations. Based on this classification, EC patients were classified into one of four molecular subtypes: *POLE* mutated (*POLE*mut), mismatch repair deficient (MMRd), p53 abnormal (p53abn), and no specific molecular profile (NSMP) (6, 7). To simplify the analysis of molecular subgroups of EC in a single exam with a single sample, the Canadian group that created the ProMisE methodology carried out a study comparing the standard method with next-generation sequencing methodology (ProMisE NGS). Of the 164 samples tested in this study, there was disagreement in only 5, with an overall accuracy of 0.97 (8).

In 2021, the European Societies of Gynaecological Oncology, Radiotherapy, and Pathology (ESGO/ESTRO/ESP) published a consensus where, for the first time, the ProMisE molecular classification began to be considered for the therapeutic management of patients with EC (9). Based on this, patients in the p53abn subgroup, even in less advanced stages, are considered high risk and undergo an upgrade in their risk stratification. In contrast, patients in the *POLE*mut subgroup, who have suffered downgrades in their risk stratification, may have a de-escalation in their adjuvant treatment (9). Moreover, ProMisE was included in the new FIGO staging published in 2023 (10). Therefore, the molecular classification will become part of the therapeutic definition of patients with EC, even more so with the future results of phase 3 studies that base their treatment protocols on this novel stratification (11, 12). Recently, the lack of diversity in genomics studies has been highlighted, leading to unmet scientific needs and health disparities (13). In this context, the knowledge of endometrial cancer molecular profile in the Latin-American population, particularly the Brazilian, is scarce.

The present study aimed to describe the implementation of the ProMisE classification for routine endometrial cancer evaluation in a Brazilian tertiary public cancer center hospital, to compare it with a molecular approach of MSI and *TP53* mutation, and to evaluate ProMisE impact on patients’ prognosis.

## 2 Methods

### 2.1 Patients

This prospective cohort study of EC attended and followed at the Gynecologic Oncology Department at Barretos Cancer Hospital (BCH), between October 2020 and December 2022. A REDCap database was created with pertinent clinical-pathological and molecular features (14). The Institutional Review Board approval was obtained from the BCH Office of Protocol Research (65587622.7.0000.5437).

The medical records of all patients who underwent total hysterectomy were prospectively reviewed. The information collected included demographics, surgical–pathology reports, and clinical outcomes. We included patients aged 18 or older diagnosed with low, intermediate, and high-risk EC, treated by surgery or systemic treatment. Exclusion criteria included patients treated surgically outside the institution referred to ours for adjuvant treatment and/or follow-up without a treatment registry or incomplete medical records. All patients underwent a pretreatment evaluation, including a physical examination, pelvic magnetic resonance imaging (MRI), and chest and abdomen computed tomography (CT). Primary surgery included a total hysterectomy and bilateral salpingo-oophorectomy. Sentinel lymph node mapping and/or systematic lymphadenectomy (pelvic and/or para-aortic) were performed according to the pre-operative data and/or intraoperative frozen section analysis. Adjuvant treatment was given at the discretion of a multidisciplinary conference. The follow-up data were obtained from clinic visits, every three months in the first two years, and every six months during years 3 to 5. Patients with high-risk EC do chest and abdominal computed tomography every 12 months in the first two years. Patients who were no longer being followed clinically by BCH were contacted by the Institution’s Department of Research to obtain information about cancer status and general medical problems. The patients were characterized into the prognostic risk groups (low, intermediate, high-intermediate, high, and advanced metastatic) according to the 2020 ESGO/ESTRO/ESP guidelines (9). All cases were histologically reviewed by GRT, MTR, and FFB, confirming the initial diagnosis (**Supplementary Figure 1**).

### 2.2 ProMisE classification

The ProMisE molecular profile was performed by Talhouk et al. (15), using MMR immunohistochemistry (IHC), followed by *POLE* hotspot mutation of the MMR proficient cases, and then p53 IHC of the POLE wildtype cases, leading to the following classification: MMRd, *POLEmut*, p53abn, and NSMP.

#### 2.2.1 Mismatch repair and p53 immunohistochemistry

Formalin-fixed paraffin-embedded (FFPE) tissue blocks were cut into 3 μm sections for IHC. For MMR enzymes, the Dako EnVision™ FLEX detection system Kit (Dako, Glostrup, Denmark) and Autostainer Link 48 equipment (Dako, Glostrup, Denmark) were used as previously described (16). The antigen retrieval process was done at 97°C for 20 minutes (pH 9.0). Endogenous peroxidases were blocked with EnVision™ FLEX Peroxidase-Blocking Reagent (Dako, Glostrup, Denmark). The primary antibodies used were: FLEX monoclonal mouse anti-MutL protein homolog 1 (MLH1) (clone ES05, ref IS079, Dako, Glostrup, Denmark); FLEX monoclonal mouse anti-MutS protein homolog 2 (MSH2) (clone FE11, ref IR085, Dako, Glostrup, Denmark); FLEX monoclonal rabbit anti-postmeiotic segregation increased 2 (PMS2) (clone EP51, ref IR087, Dako, Glostrup, Denmark); and FLEX monoclonal rabbit anti-MutS protein homolog 6 (MSH6) (clone EP49, ref IR086, Dako, Glostrup, Denmark). The DAB solution was used for immunostaining visualization. Slides were counterstained with hematoxylin. In each case, nuclear staining of normal epithelial cells, lymphocytes, and stromal cells served as positive internal controls. All cases were analyzed by expert pathologists (GRT, MTR, and FFB) who, based on nuclear staining, classified each protein by its expression. Regardless of the intensity or the extent of cell staining, the positive status was found for the cases that showed staining (presence of the expression of the protein under analysis) and the negative status when no staining was present (absence of expression of the protein under analysis) in tumor cells.

For p53, immunostaining was performed using the BenchMark Ultra platform (Ventana Medical System, Arizona, USA) with the UltraView^®^ signal detection kit (Ventana Medical System, Arizona, USA). The primary antibody was anti-p53 (clone DO-7, Roche Diagnostics, Indiana, USA). Immunoexpression was considered abnormal when it was positive, strong, and diffuse in more than 80% of tumor cells (positive pattern) or entirely negative with positive intern control (null pattern), as reported (17).

#### 2.2.2 *POLE* mutation detection

Tumor FFPE DNA was isolated using QIAamp DNA Micro Kit (Qiagen, Hilden, Germany) as previously reported (18). The hotspot exonuclease domain of *POLE* (exons 9-14) was evaluated by Sanger sequencing. Polymerase chain reaction (PCR) was performed using M13-tailed primers as described elsewhere (19). The PCR products were purified using Exo+Sap (Cellco), followed by bidirectional sequencing with universal M13 tags and the BigDye Terminator v3.1 Cycle Sequencing Kit (Thermo Fisher Scientific). Capillary electrophoresis was carried out on an ABI 3500xL Genetic Analyzer (Applied Biosystems), and the resulting chromatograms were analyzed using SeqScape software v3.0 (Applied Biosystems) in addition to manual inspection.

### 2.3 ProMisE molecular approach

MMR and *TP53* status were molecularly evaluated. For MMR, a PCR-based approach was used with the HT-MSI+ Kit (Cellco), which comprises six repeat markers (NR27, NR21, NR24, BAT25, BAT26, and HSP110), following the manufacturer’s instructions. Capillary electrophoresis was performed on an ABI 3500 Genetic Analyzer (Applied Biosystems), and the results were analyzed using GeneMapper v4.1 software (Applied Biosystems). Cases demonstrating the presence of two or more markers falling outside the quasimonomorphic variation range (QMVR) were categorized as MSI-H (high microsatellite instability), while cases without markers outside the QMVR were classified as MSS (microsatellite stable) (20).

The mutational analysis of *TP53* coding region was assessed by NGS using the TruSight Tumor 15 Panel (Illumina) on the MiSeq System (Illumina). The analysis was carried out in the Sophia DDM^®^ software v4.2 (Sophia Genetics). Variants were filtered out according to the following criteria: intronic (except splicing variants), synonymous singles nucleotide variants (SNVs), populational frequency >1%, poor quality of the read depth <500x, allele frequency <10%, and non-pathogenic variants (21).

### 2.4 Statistical analysis

Both quantitative and qualitative variables were initially explored using descriptive statistics. Quantitative variables were presented as mean and standard deviation or median and 25-75 percentiles, according to the data distribution measured by the Kolmigorov-Smirnov test. Qualitative variables were described using absolute and relative frequencies. Statistical analysis was carried out using Student’s T-test if the sample had a normal distribution or McNemar’s test if the sample was non-parametric. For categorical variables, the Chi-square or Fisher’s Exact test were used to compare the proportions between groups. When appropriate, 95% confidence intervals were calculated, for detecting a true effect considered a reasonable threshold in fields of research with risk of falsely decteing (type II error). The multivariate Cox regression model was analyzed to interpret prognostic information that can complement the molecular classification (demographic, surgical–pathology characteristics, and oncology outcomes). The Kappa coefficient was used to assess the concordance and accuracy between both methods evaluated (ProMise and ProMisE molecular approach).

Overall survival (OS) and progression-free survival (PFS) were estimated using the Kaplan-Meier method along with the Log-rank test, stratified by molecular classification. If necessary, we used Cox regression analysis techniques. Overall survival was defined as the time between the initial diagnosis and the date of the patient’s death from any cause or last contact. Progression-free survival was defined as the time between the initial diagnosis and the date on which disease progression was proven or the last contact. The data was analyzed using SPSS (Statistical Package for the Social Sciences) version 21.0 and R program version 4.4.0 (2024). Statistical significance was defined as p <0.05 with probability for level false positive rate (type I error rate) provides a likelihood inference.

## 3 Results

A total of 114 EC samples were analyzed prospectively. Clinicopathologic characteristics related to ProMisE classification are shown in **Table 1**. Approximately 50% of patients were stage I, while 10.5% were metastatic at diagnosis. Sixty-eight (59.6%) patients had histological grade 3, 30 (26.3%) of which had non-endometrioid histology. Considering ESGO risk stratification, 63 (55.3%) patients were considered high-risk, supporting adjuvant treatment (**Table 1**).

**Table 1:** Clinicopathologic features of the total cohort by ProMisE classifier.

	Total	*POLE*mut	MMRd	p53abn	NSMP	p value
**Total (%)**	114 (100)	18 (15.8)	32 (28.1)	31 (27.2)	33 (28.9)	
**Age (mean)^a^**	63.1 ( ± 9.8)	57.7 ( ± 5.8)	63.5 ( ± 9.8)	66.9 ( ± 6.0)	62.0 ( ± 6.0)	**< 0.001**
**BMI (mean)^a^**	30.7 ( ± 7.2)	30.9 ( ± 6.6)	30.7 ( ± 6.8)	28.9 ( ± 6.9)	32.0 ( ± 8.0)	**0.2**
**Figo Stage (%)**						**0.015**
IA	28 (24.6)	5 (27.8)	12 (37.5)	4 (12.9)	7 (21.2)	
IB	28 (24.6)	8 (44.4)	6 (18.8)	6 (19.3)	8 (24.2)	
II	8 (7.0)	2 (11.1)	2 (6.2)	0 (0.0)	4 (12.1)	
IIIA	10 (8.8)	1 (5.6)	3 (9.4)	2 (6.5)	4 (12.1)	
IIIB	4 (3.5)	0	1 (3.1)	2 (6.5)	1 (3.0)	
IIIC	24 (21.0)	2 (11.1)	8 (25.0)	11 (35.5)	3 (9.1)	
IVB	12 (10.5)	0	0	6 (19.3)	6 (18.3)	
**Tumor Grade (%)**						**< 0.001**
1	5 (4.4)	0 (0.0)	2 (6.3)	0 (0.0)	3 (9.1)	
2	41 (36.0)	7 (38.9)	13 (40.6)	3 (9.7)	18 (54.5)	
3	68 (59.6)	11 (61.1)	17 (53.1)	28 (90.3)	12 (36.4)	
**Histological Subtype (%)**						**< 0.001**
Endometrioid	84 (73.7)	15 (83.3)	31 (96.9)	9 (29.0)	29 (87.9)	
Serous	12 (10.5)	0 (0.0)	0 (0.0)	10 (32.3)	2 (6.1)	
Clear cell	4 (3.5)	1 (5.6)	1 (3.1)	1 (3.2)	1 (3.0)	
Carcinosarcoma	9 (7.9)	1 (5.6)	0 (0.0)	7 (22.6)	1 (3.0)	
Mixed/Undifferentiated	5 (4.4)	1 (5.6)	0 (0.0)	4 (12.9)	0 (0.0)	
**LVSI (%)**						**0.066**
Negative	52 (45.6)	11 (61.1)	15 (46.9)	8 (25.8)	18 (54.5)	
Positive	57 (50.0)	7 (39.9)	17 (53.1)	20 (64.5)	13 (39.4)	
Missing	5 (4.4)	0 (0.0)	0 (0.0)	3 (9.7)	2 (6.1)	
**ESGO 2021 Risk Classification (%)**						**0.001**
Low	11 (9.6)	3 (16.7)	5 (15.6)	0 (0.0)	3 (9.1)	
Intermediate	16 (14.0)	5 (27.8)	3 (9.4)	3 (9.7)	5 (15.1)	
High-Intermediate	24 (21.1)	4 (22.2)	11 (34.4)	0 (0.0)	9 (27.3)	
High	63 (55.3)	6 (33.3)	13 (40.6)	28 (90.3)	16 (48.5)	
**Lymph node status (%)**						**0.036**
Negative	71 (62.3)	16 (88.9)	22 (68.8)	12 (38.7)	21 (63.6)	
Positive	25 (21.9)	2 (11.1)	8 (25.0)	11 (35.5)	4 (12.1)	
Unperformed	18 (15.8)	0 (0.0)	2 (6.2)	8 (25.8)	8 (24.3)	
**Adjuvant treatment (%)**						**0.034**
None	16 (14.0)	3 (16.7)	7 (21.9)	0 (0.0)	6 (18.2)	
Radiotherapy	45 (39.5)	11 (61.1)	12 (37.5)	8 (25.8)	14 (42.4)	
Radiotherapy + chemotherapy	34 (29.8)	4 (22.2)	9 (28.1)	14 (45.2)	7 (21.2)	
Chemotherapy	12 (10.5)	0 (0.0)	3 (9.4)	5 (16.1)	4 (12.1)	
Unperformed (Stage IV)	7 (6.2)	0 (0.0)	1 (3.1)	4 (12.9)	2 (6.1)	
**Recurrence (%)**						**0.003**
No	84 (73.7)	18 (100)	25 (78.1)	17 (54.8)	24 (72.7)	
Yes	30 (26.3)	0 (0.0)	7 (21.9)	14 (45.2)	9 (27.3)	
**Local recurrence (%)**						**0.083**
No	94 (82.5)	18 (100)	25 (78.1)	23 (74.2)	28 (84.8)	
Yes	20 (17.5)	0 (0.0)	7 (21.9)	8 (25.8)	5 (15.2)	
**Systemic recurrence (%)**						**0.013**
No	96 (84.2)	18 (100)	30 (93.8)	22 (71.0)	26 (78.8)	
Yes	18 (15.8)	0 (0.0)	2 (6.2)	9 (29.0)	7 (21.2)	
**Disease status (%)**						**0.006**
Alive without recurrence	79 (69.3)	17 (94.4)	23 (71.9)	14 (45.2)	25 (75.8)	
Alive with recurrence	17 (14.9)	0 (0.0)	5 (15.6)	7 (22.6)	5 (15.1)	
Death from câncer	14 (12.3)	0 (0.0)	2 (6.2)	9 (29.0)	3 (9.1)	
Unknown death	4 (3.5)	1 (5.6)	2 (6.2)	1 (3.2)	0 (0.0)	

BMI, body mass index; MMRd, mismatch repair deficient; NSMP, no specific molecular profile; *POLE*mut, *POLE* mutated; p53abn, p53 abnormal; LVSI, lymphovascular space invasion. a, Mann-Whitney test; b, Fisher’s exact test. Bold, significant values.

Evaluating ProMisE classification (**Table 1**), we observed 18 (15.8%) *POLE*mut (**Supplementary Figure 2**), 32 (28.1%) MMRd (**Supplementary Figure 3**), 31 (27.2%) p53abn (**Supplementary Figure 4**), and 33 (28.9%) NSMP EC. There was a statistical difference between the groups in age, FIGO stage, tumor grade, histological subtype, ESGO risk classification, lymph node status, recurrence, and disease status. Namely, the recurrence rate, a significant unfavorable association was found for the p53abn and NSMP subgroup (*p=*0.013) (**Table 1**). Furthermore, the cancer mortality rate was more prevalent in the p53abn subgroup, with 29.0% of deaths (*p=*0.006) (**Table 1**).

Next, we compared the classification of the traditional ProMisE with the molecular approach (**Table 2**). Among the 114, we had 10 (8.7%) with discordant results between the MMR and MSI status (**Supplementary Figure 5** and **Table 3**). Eight cases depicted the loss of one or more MMR proteins but showed an MSS phenotype, and two cases displayed an MSI-H status despite the expression of all MMR proteins (**Table 3**). Concerning *TP53* status, among the 114, we observed 31 (27.2%) mutated cases (**Supplementary Figure 6**) and found six discordances methodologies (**Table 4**). Overall, the traditional and molecular PROMISE approaches showed a concordance rate of 86.8% (99/114 cases) with an overall accuracy of 0.87 and a Kappa coefficient of 0.82 (**Table 5**).

**Table 2:** Prevalence of subgroups by ProMisE and molecular classification.

Molecular classifier	ProMisE (%)	Molecular (%)
*POLE*mut	18 (15.8)	18 (15.8)
MMRd/MSI	32 (28.1)	27 (23.7)
p53abn/*TP53*mut	31 (27.2)	31 (27.2)
NSMP/*TP53*wt	33 (28.9)	38 (33.3)
Total	114 (100)	114 (100)

ProMisE, Proactive Molecular Risk Classifier for Endometrial Cancer; MMRd, mismatch repair deficient; MSI, microsatellite instability; NSMP, no specific molecular profile; *POLE*mut, *POLE* mutated; p53abn, p53 abnormal; *TP53*mut, *TP53* mutated; *TP53* wt, *TP53* wild-type. Accuracy, 0.87; Kappa, 0.82.

**Table 3:** Discordant MMR and MSI cases.

MSS status	MMR immunohistochemistry			
	MSH2	MSH6	MLH1	PMS2
ID29	Positive	Positive	Negative	Negative
ID30	Positive	Negative	Positive	Positive
ID34	Positive	Positive	Negative	Negative
ID37	Negative	Negative	Positive	Positive
ID62	Positive	Positive	Negative	Negative
ID90	Positive	Negative	Positive	Positive
ID98	Positive	Positive	Negative	Negative
ID187	Positive	Positive	Negative	Negative
ID29	Positive	Positive	Negative	Negative
ID30	Positive	Negative	Positive	Positive
ID34	Positive	Positive	Negative	Negative
MSI-H status				
ID93	Positive	Positive	Positive	Positive
ID163	Positive	Positive	Positive	Positive

MMR, mismatch repair; MSS, microsatellite stable, MSI-H, high microsatellite instability.

The red color indicates the disagreement between the two methodologies used.

**Table 4:** Discordant p53 IHC and TP53 NGS cases.

	*TP53* NGS	p53 status
ID31	p.(His179Arg) and p.(Tyr236Thrfs*98)	wild-type expression
ID49	p.(Ile255del)	wild-type expression
ID51	p.(Arg248Trp)	wild-type expression
ID58	wild-type	abnormal expression
ID101	wild-type	abnormal expression
ID183	wild-type	abnormal expression

**Table 5:** Shifting of cases between molecular profiles using ProMisE and molecular classifier.

Molecular classifier	ProMisE classifier (%)				
	*POLE*mut	MMRd	p53abn	NSMP	Total
*POLE*mut	**18 (100)**	0	0	0	**18**
MSI	0	**25 (78.1)**	0	2 (6.1)	**27**
*TP53*mut	0	0	**28 (90.3)**	3 (9.1)	**31**
*TP53*wt	0	7 (21.9)	3 (9.7)	**28 (84.8)**	**38**
**Total**	**18**	**32**	**31**	**33**	**114**

ProMisE, Proactive Molecular Risk Classifier for Endometrial Cancer; MMRd, mismatch repair deficient; MSI, microsatellite instability; NSMP, no specific molecular profile; *POLE*mut, *POLE* mutated; p53abn, p53 abnormal; *TP53*mut, *TP53* mutated; *TP53*wt, *TP53* wild-type.

Bold values indicates the agreement between the two methodologies used.

**Figure 1:**
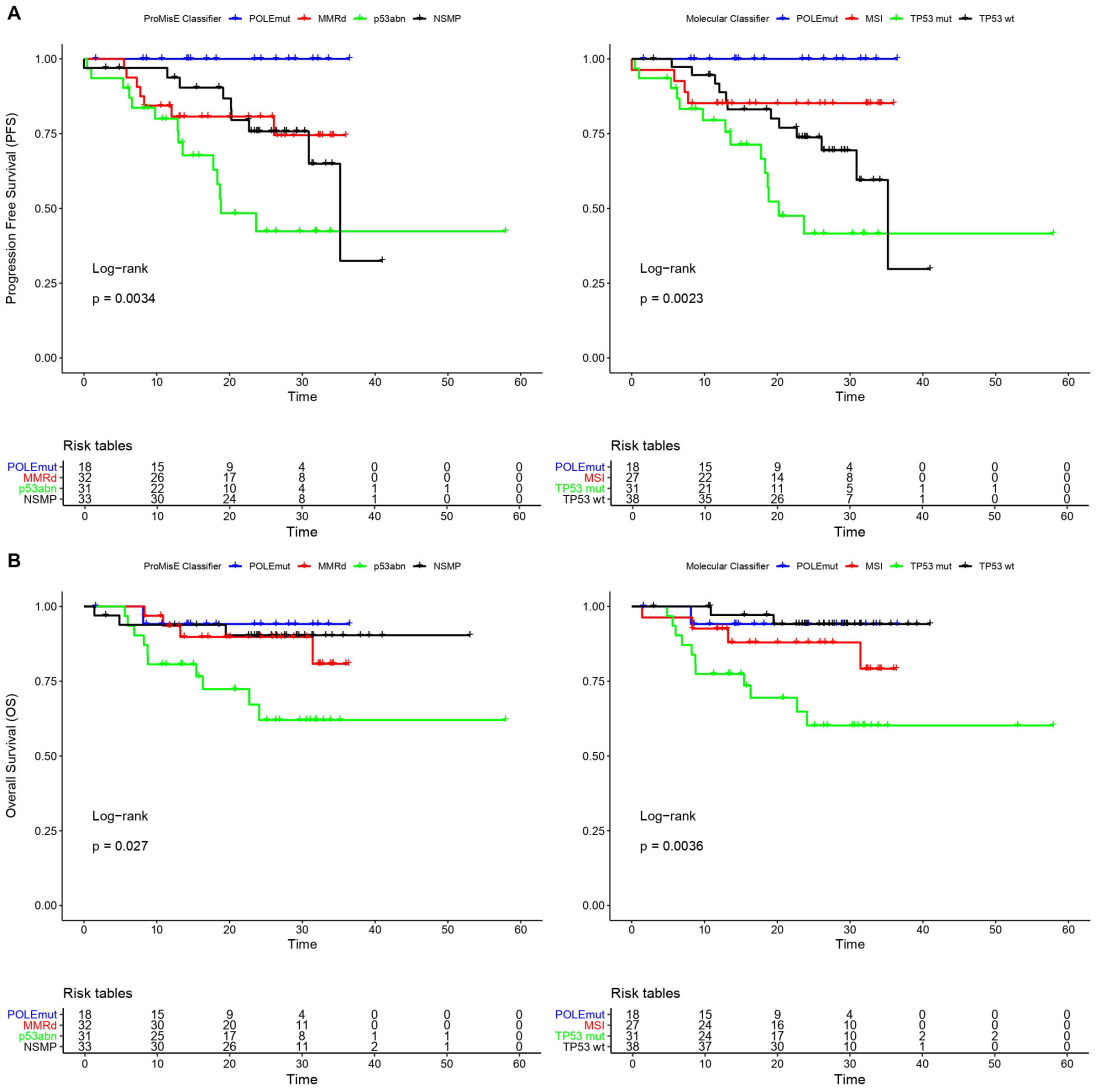
Kaplan-Meier survival analyses of both methods evaluated (ProMisE and ProMisE molecular approach). **(A)** Progression-free survival. **(B)** Overall survival.

We further evaluated the prognostic impact of ProMisE classification, both traditional and molecular approaches. After a median follow-up of 23.2 months (± 10.6 months), a statistically significant difference was observed in terms of OS (p=0.027) and PFS (p=0.0034) related to the four molecular subtypes using the ProMisE classifier (**Figure 1** and **Table 6**). Assessed by molecular classification, both outcomes maintain statistically significant differences with curves very similar to those of the traditional methodology (OS: p = 0.0036; PFS: p = 0.0023) (**Figure 1** and **Table 6**). In this prospective cohort, attention is drawn to the excellent overall survival not only of the *POLE*mut subgroup, but also of MMRd and NSMP.

**Table 6:** Overall survival (OS) and progression-free survival (PFS) outcomes in both methodologies (24 months of follow-up).

Molecular classifier	PFS (%)	OS (%)
*POLE*mut	100	93.8
MSI	82.6	85.4
*TP53*mut	48.7	66.1
*TP53*wt	70.3	95.0
ProMisE classifier	PFS (%)	OS (%)
*POLE*mut	100	93.8
MMRd	75.4	88.1
p53abn	48.3	68.9
NSMP	74.1	91.4

ProMisE, Proactive Molecular Risk Classifier for Endometrial Cancer; MMRd, mismatch repair deficient; MSI, microsatellite instability; NSMP, no specific molecular profile; *POLE*mut, *POLE* mutated; p53abn, p53 abnormal; *TP53*mut, *TP53* mutated; *TP53*wt, *TP53* wild-type.

## 4 Discussion

In the present study, we implemented the ProMisE classifier in a Brazilian prospective cohort of patients with EC from a tertiary cancer hospital. Furthermore, we proposed comparing this traditional methodology with a molecular methodology to evaluate its accuracy and reproducibility.

We observed that the proportion of patients in each of the four subgroups considering the ProMisE classifier was 15.8% *POLE*mut, 28.1% MMRd, 27.2% p53abn, and 28.9% NSMP. Overall, our findings align with the range reported in populations, namely from North America, Europe, Asia, and Oceania, which showed prevalence rates of these subgroups of 4-15% *POLE*mut, 17-38% MMRd, 9-25% p53abn, and 40-64% NSMP (6, 8, 14, 22–25). Proportionally, we had fewer cases in the NSMP subgroup and slightly higher cases in the p53abn subgroup. Further studies are needed to elucidate these differences, mainly the higher frequency of p53abn cases. Nevertheless, we can hypothesize that it could be due to the reference nature of our hospital, which receives cases with more advanced diagnoses (26). Additionally, it could be related to distinct exposure or the admixture ethnicity of the Brazilian population, as we recently reported the higher frequency of *TP53* mutations in lung cancer patients associated with African ancestry (21). Notably, the p53abn subgroup classification was a significant factor for both PFS and OS, exhibiting the poorest outcomes.

Considering the histological subtypes and their molecular profile, we had 84 patients (73.7%) with endometrioid histology, 17.9% of these in the *POLE*mut subgroup, 36.9% in the MMRd subgroup, 10.7% in the p53abn subgroup, and 34.5% in the NSMP subgroup. The other 30 patients (26.3%) had non-endometrioid histologies (serous, clear cell, carcinosarcoma), with a prevalence of molecular profile represented as follows: 10.0% *POLE*mut, 3.3% MMRd, 73.4% p53abn, and 13.3% NSMP subgroup. Compared with TCGA data, the enormous prevalence of patients with *p53* mutation is represented by non-endometrioid histologies, and less than 5% of patients with *POLE* mutation are in this histological subgroup. The present cohort has less than a third of patients when compared to the TCGA study, which makes it difficult to perform more detailed subgroup analyses such as that carried out in the pivotal study (low-grade endometrioid *versus* high-grade endometrioid; serous *versus* clear cell *versus* carcinosarcoma) (5, 27). Further studies in a more extensive Brazilian series of endometrioid and non-endometrioid histologies are warranted to compare the differences in molecular subgroups among them.

In our study, the concordance and accuracy comparing ProMisE and molecular classifiers were 86.8% and 87%, respectively. Two studies, one Canadian led by Jessica McAlpine (8), and the other Chinese led by Jinaliu Wang (28), evaluated the comparison between two methodologies, the traditional ProMisE classifier and another using next-generation sequencing (NGS) molecular classifier (ProMisE NGS) and found slightly higher values, probably due to higher number of cases assessed. The Canadian study identified a concordance rate of 97% (159/164), whereas in the Chinese study, this rate was 94.1% (451/479) (8, 28). Concerning the MSI, we evaluated using a PCR-based approach previously validated at BCH for distinct tumor types (18, 20, 29, 30). The concordance rate was 86.8% (99/114) and is in accordance with reported discrepancies in methodological agreement in endometrial cancer. Dedeurwaerdere and colleagues evaluated three different molecular techniques, including one using PCR, to compare with immunohistochemistry results to define colorectal and EC patients with MSI. The concordance rate for patients with colorectal cancer was 100%, while for patients with EC, it ranged from 58% to 75% (31). A Spanish study also evaluated different methodologies for defining MMR status (IHC, PCR, NGS) in EC. The results showed discordance rates among the three techniques (32), which in part could be due to the known discordant behavior of the MSH6 pattern loss (33). McConechy et al. demonstrated a 93.3% concordance rate comparing pentaplex mono and di-nucleotide PCR tests for MSI with MMR status by IHC, suggesting that further studies are needed to define the best diagnostic technique for MMRd in EC (34).

The concordance rate for p53 by IHC and *TP53* sequencing by NGS was 90.3%, similar to other reports. Singh et al. conducted a study with just over 200 patients, comparing p53 status by IHC and *TP53* sequencing by NGS, demonstrating an overall agreement rate of 92.3% (35). Another study evaluating patients with high-risk EC in the PORTEC-3 trial reported a 90.7% concordance rate between p53 IHC and *TP53* NGS analysis (36).

Although the ProMisE methodology has been well documented internationally (6), there has never been a report of its use in Brazilian endometrial cancer patients. Furthermore, we conducted a second classification, based only on molecular methodologies, and carried it out in a public hospital in Brazil. Additionally, this is a prospective cohort where we included all cases referred for treatment at the BCH, thus minimizing selection bias that could influence the results demonstrated. Of note, the entire therapeutic definition of the patients was discussed in a weekly tumor board of the gynecologic oncology department of the aforementioned hospital, thus minimizing possible influences on the oncological outcomes of these patients.

Despite our study’s significant findings and relevance, it also exhibits some limitations. The number of patients included is a limiting factor for a large and significant statistical association, as is the short follow-up time to define oncological outcomes in patients with EC. Furthermore, the molecular approach using distinct methodologies is not ideal, and we are currently working on a single NGS assay to assess all the biomarkers of the ProMisE classifier.

## 5 Conclusion

In this prospective cohort of endometrial cancer treated at a single public institution in Brazil, we successfully implemented the ProMisE classification for all patients and demonstrated its impact on stratifying patients’ outcomes. The molecular subgroups were in line with the international literature, with a slight increase in the proportion of *TP53* mutated patients. Furthermore, with the current development of more comprehensive NGS panels, with the inclusion of *POLE*, *TP53* genes, MSI, and other putative actionable endometrial genes, we foresee that a single NGS methodology would be the most effective, quick, objective, and cost-effective approach for endometrial cancer molecular classification.

## Data Availability

The original contributions presented in the study are included in the article/**Supplementary Material**. Further inquiries can be directed to the corresponding author.

## Supplementary material

Supplementary Figure 1 Illustrative cases of endometrioid carcinomas with different molecular subtypes (20X objective). **(A)** No specific molecular profile (NSMP); **(B)** p53 abnormal (p53abn); **(C)** POLE mutated (*POLE*mut); and **(D)** Mismatch repair deficient (MMRd).

Supplementary Figure 2 Sanger sequencing electropherogram of hotspot *POLE* mutations.

Supplementary Figure 3 An illustrative case of mismatch repair proteins by immunohistochemistry. **(A, B)** reveal MSH2 and MSH6 intact nuclear expression by tumor cells, as **(C, D)** reveal PMS2 and MLH1 loss of nuclear expression, respectively (20X objective).

Supplementary Figure 4 Illustrative cases with P53 abnormal immunoexpression: overexpression in **(A)**, demonstrated by strong and diffuse nuclear positivity in more than 90% of tumor cells; null pattern in **(B)**, lack of nuclear or cytoplasmic expression in tumor cells (10X objective).

Supplementary Figure 5 Molecular fragment analysis for MSI with hexaplex panel marker. **(A)** Sample with presence of MSI (MSI-H). **(B)** Sample with absence of MSI (MSS). Arrow indicates the allele outside of the QMVR (gray zone) demonstrating instability.

Supplementary Figure 6 Molecular analysis of *TP53* in endometrial cancer patients. **(A)** Sequenced reads of a *TP53* mutation depicted in Integrative Genomics Viewer (IGV) tool. **(B)** Sanger sequencing electropherogram of a *TP53* mutation previously identified in NGS test. **(C)** Lollipop plot of all *TP53* mutations identified in EC patients.
