# Supplementary figures and images for: Implementation of the ProMisE classifier and validation of its prognostic impact in Brazilian endometrial carcinomas
Source: Front Oncol. 2024 Dec 13;14:1503901. doi: 10.3389/fonc.2024.1503901 (PMC11671357; doi:10.3389/fonc.2024.1503901)

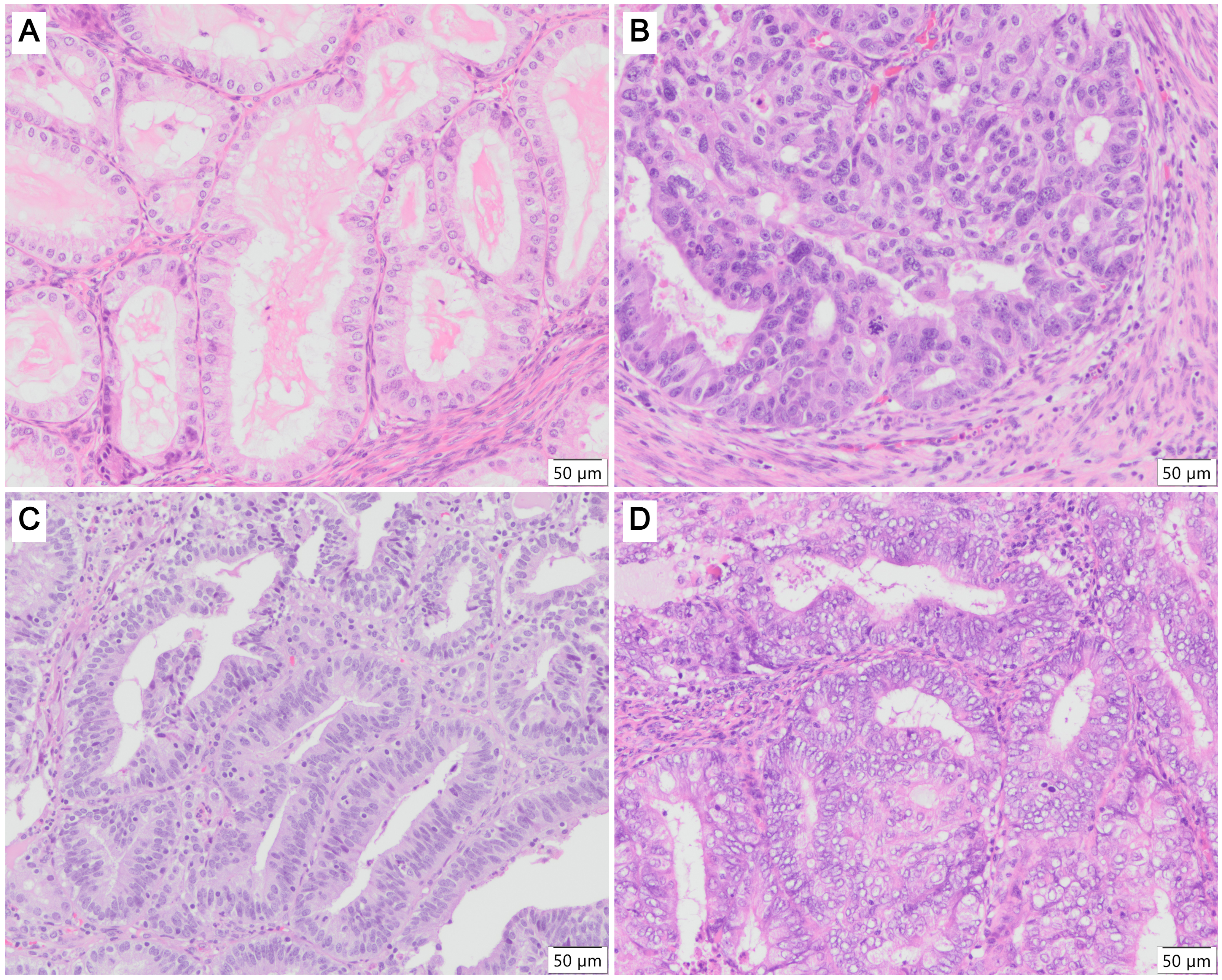

Supplement: Supplementary Figure 1 — Illustrative cases of endometrioid carcinomas with different molecular subtypes (20X objective). (A) No specific molecular profile (NSMP); (B) p53 abnormal (p53abn); (C) POLE mutated (POLEmut); and (D) Mismatch repair deficient (MMRd). [file Image1.png]

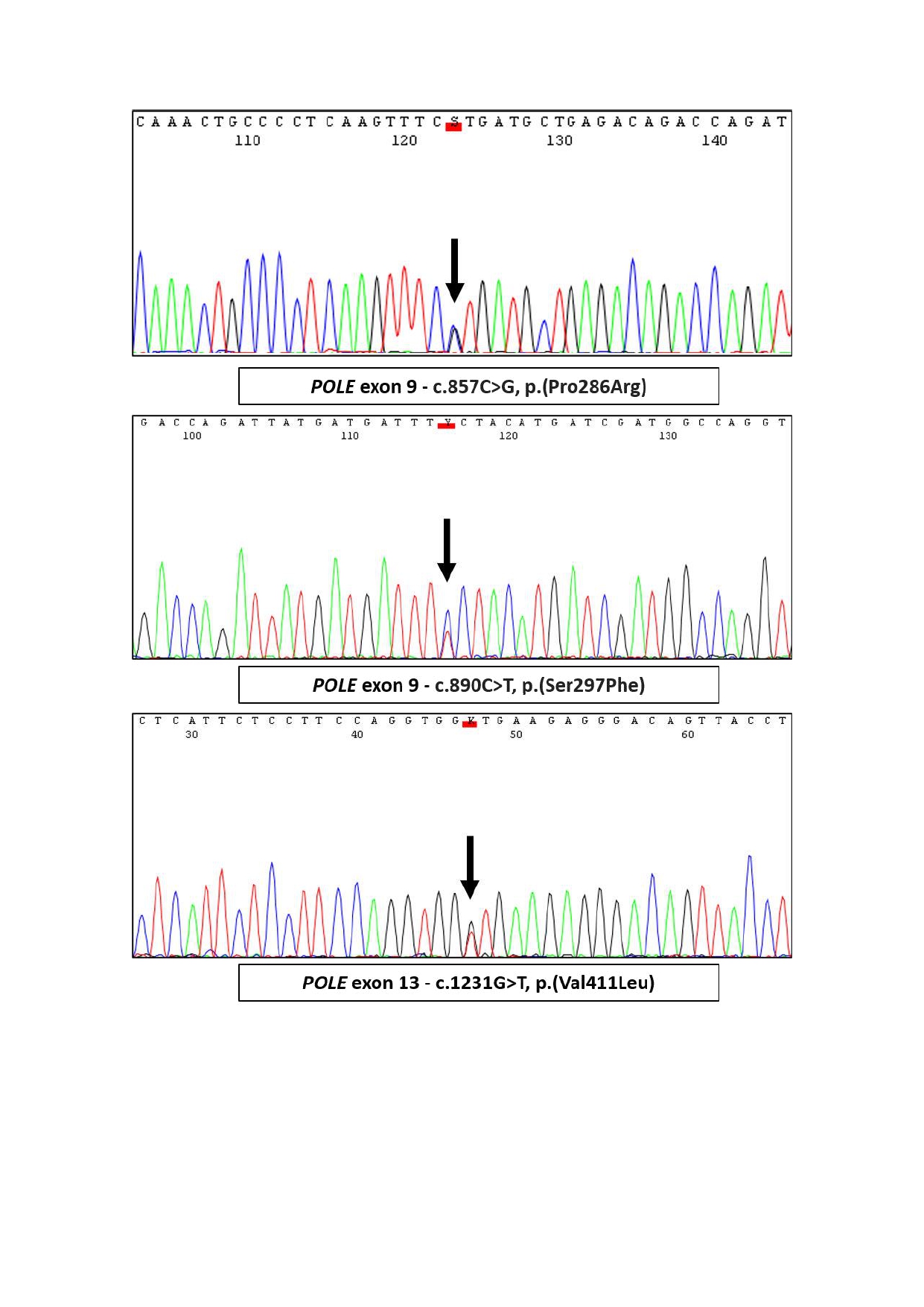

Supplement: Supplementary Figure 2 — Sanger sequencing electropherogram of hotspot POLE mutations. [file Image2.jpeg]

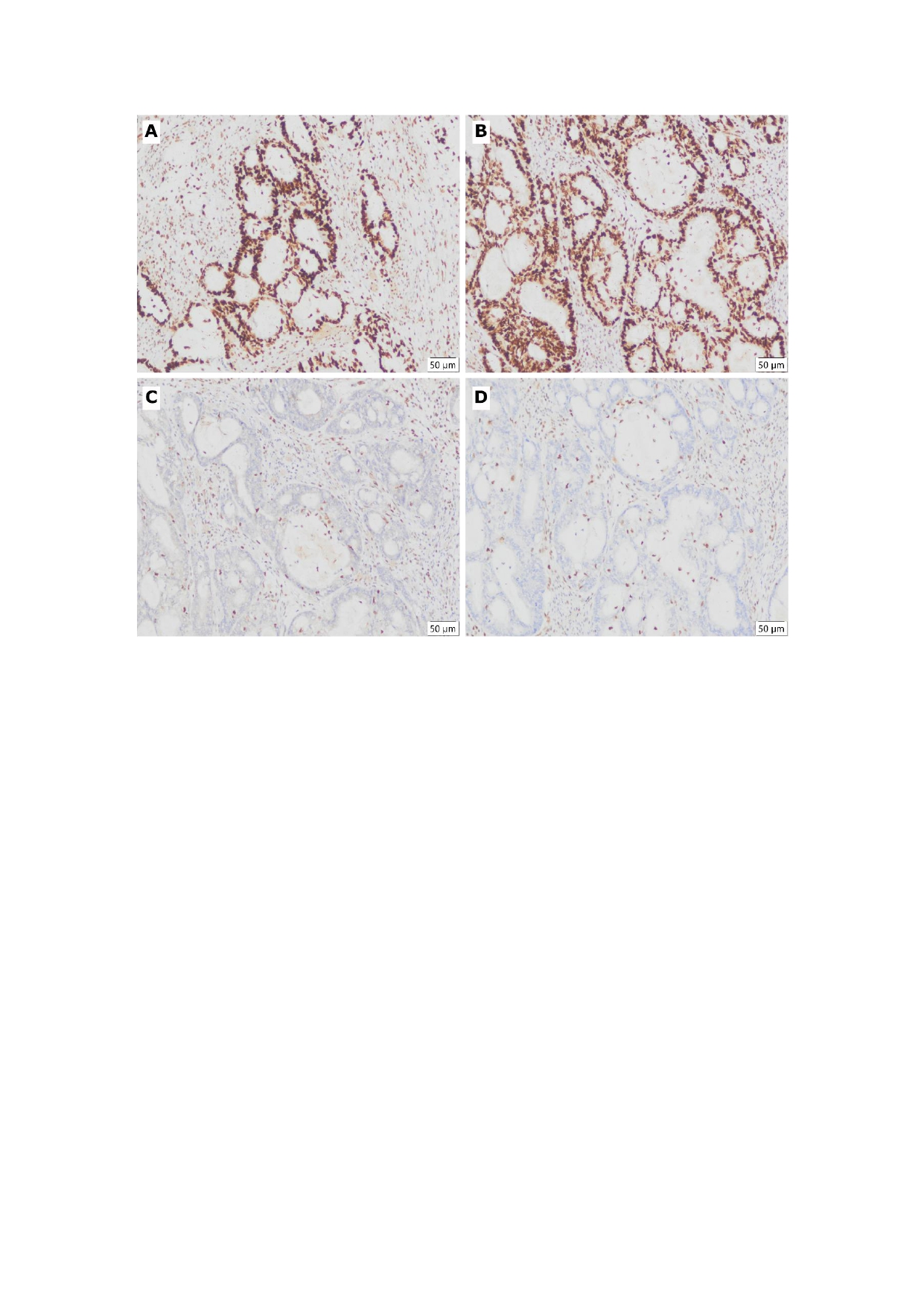

Supplement: Supplementary Figure 3 — An illustrative case of mismatch repair proteins by immunohistochemistry. (A, B) reveal MSH2 and MSH6 intact nuclear expression by tumor cells, as (C, D) reveal PMS2 and MLH1 loss of nuclear expression, respectively (20X objective). [file Image3.jpeg]

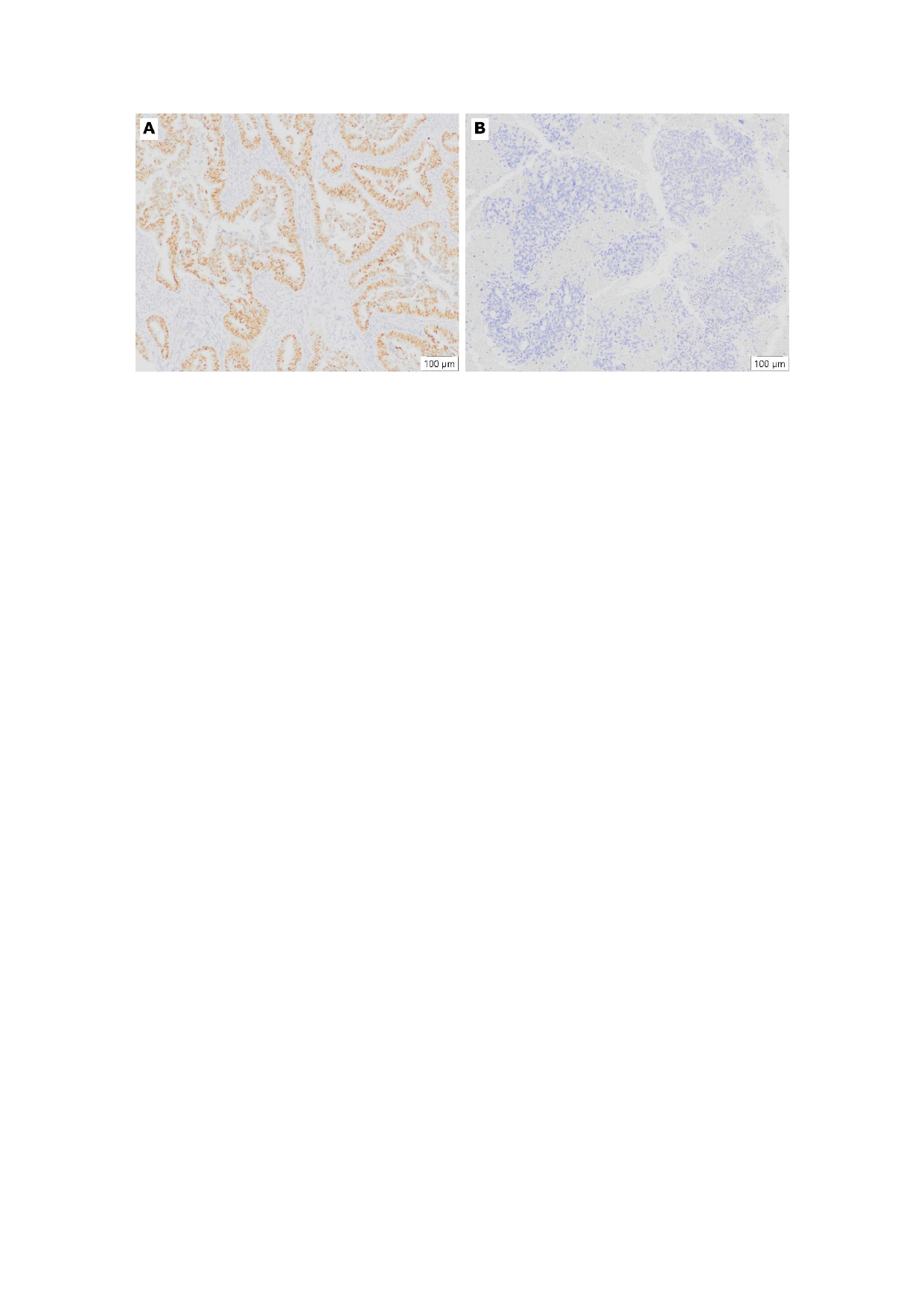

Supplement: Supplementary Figure 4 — Illustrative cases with P53 abnormal immunoexpression: overexpression in (A), demonstrated by strong and diffuse nuclear positivity in more than 90% of tumor cells; null pattern in (B), lack of nuclear or cytoplasmic expression in tumor cells (10X objective). [file Image4.jpeg]

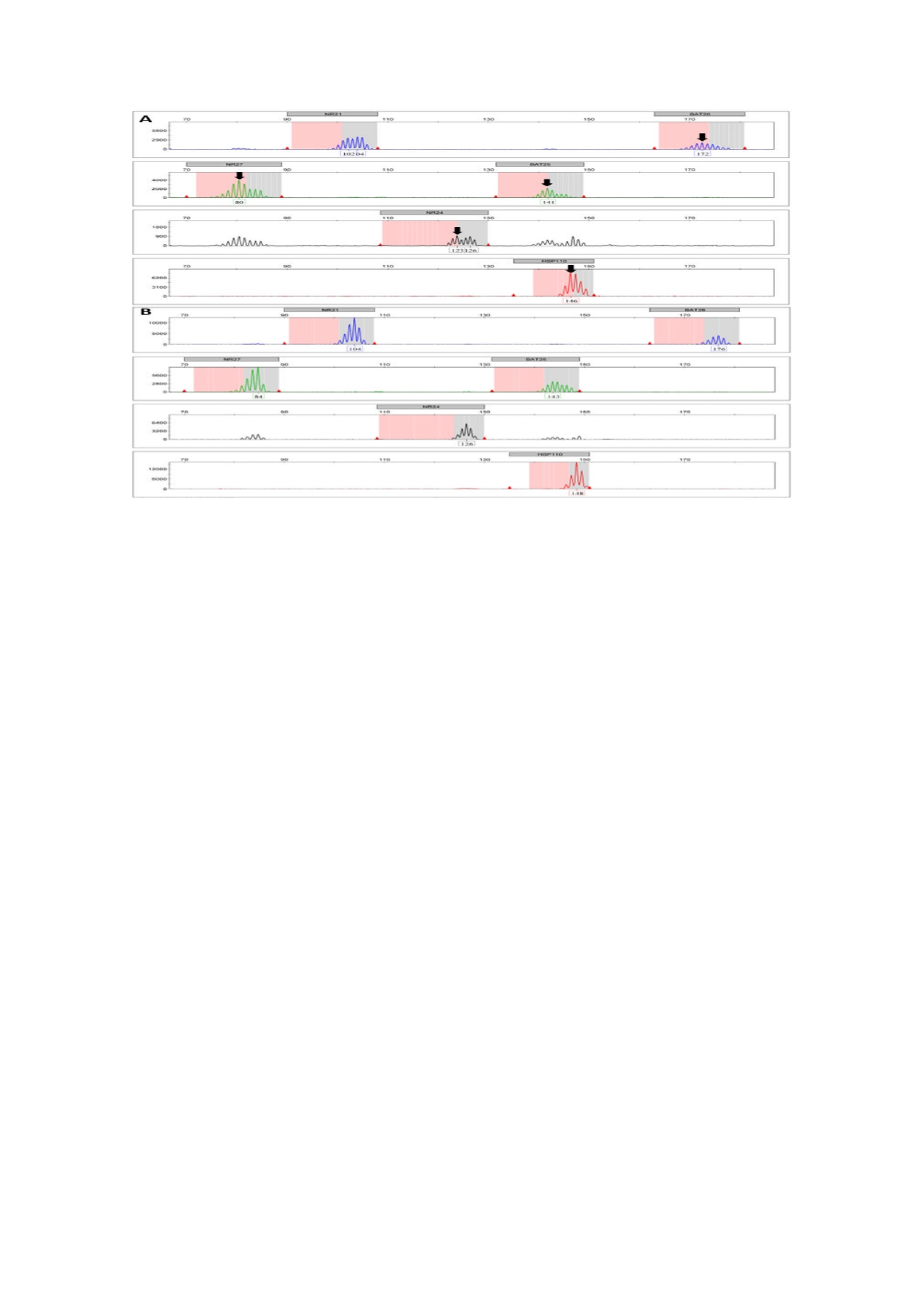

Supplement: Supplementary Figure 5 — Molecular fragment analysis for MSI with hexaplex panel marker. (A) Sample with presence of MSI (MSI-H). (B) Sample with absence of MSI (MSS). Arrow indicates the allele outside of the QMVR (gray zone) demonstrating instability. [file Image5.jpeg]

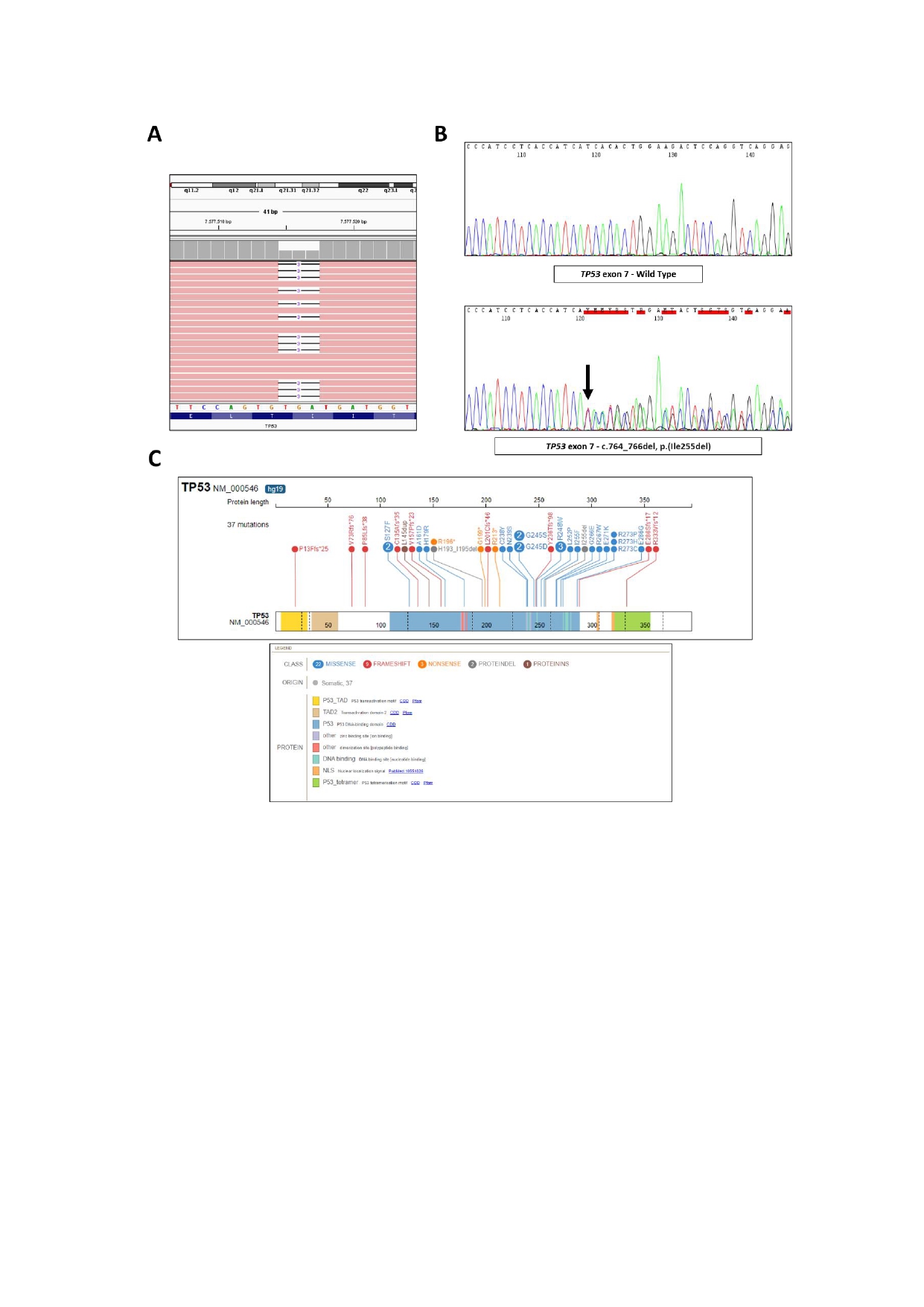

Supplement: Supplementary Figure 6 — Molecular analysis of TP53 in endometrial cancer patients. (A) Sequenced reads of a TP53 mutation depicted in Integrative Genomics Viewer (IGV) tool. (B) Sanger sequencing electropherogram of a TP53 mutation previously identified in NGS test. (C) Lollipop plot of all TP53 mutations identified in EC patients. [file Image6.jpeg]
